# Body Mass Index as a Potential Mediator of the Association Between Gout and Hypertension Among Chinese Older Adults: A Mediation Analysis in a Cross‐Sectional Study

**DOI:** 10.1002/agm2.70049

**Published:** 2025-10-13

**Authors:** Jia Wang, Jian‐bo Zhan, Lei Yi, Can Mei, Lin‐wanyue Chen, Gui‐ping Wang, Zi‐jun Shi, Wei‐ji Zhou, Chang‐e Xiong, Jing Cheng

**Affiliations:** ^1^ School of Public Health Wuhan University of Science and Technology Wuhan Hubei China; ^2^ Hubei Province Key Laboratory of Occupational Hazard Identification and Control Wuhan University of Science and Technology Wuhan Hubei China; ^3^ Hubei Provincial Center for Disease Control and Prevention Wuhan Hubei China; ^4^ Laboratory Department Huanggang Center for Disease Control and Prevention Huanggang Hubei China

**Keywords:** analysis of mediation, body mass index, cross‐sectional study, gout, hypertension

## Abstract

**Objectives:**

This study aimed to investigate the role of the body mass index (BMI) as a mediator between gout and hypertension in older adults.

**Methods:**

A total of 33,778 older adults (aged 65 years and over) in Wuhan, China, were surveyed. The propensity score matching (PSM) method was used to control for confounding factors, and logistic regression was performed on the above three variables using the mediation package in the R language program.

**Results:**

The PSM method successfully matched 14,717 pairs. Mediation analysis revealed that, when controlling for the mediating variable “BMI”, the association coefficients of the independent variable “gout” on the dependent variable “hypertension” and of the mediating variable were statistically significant. This indicates the existence of a mediating association. Bootstrapping was used to quantify the stability of the observed indirect association, but temporal precedence or causality could not be established in this cross‐sectional study.

**Conclusions:**

Gout is associated with hypertension, both directly and indirectly through BMI‐related pathways. Therefore, monitoring and controlling the BMI of elderly gout patients to improve the progression of hypertension is of great significance.

## Graphical abstract

This study employed mediation analysis and found that BMI played a significant mediating role in the relationship between gout and hypertension. The mediating effect accounted for 12.59% of the total effect (ACME = 0.0244). ACME, average causal mediation effect.

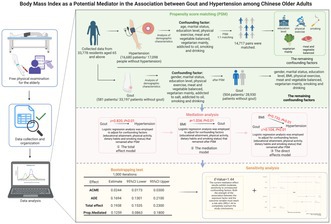

**Abbreviations**
ACME: average causal mediation association
ADE: average direct association
BMI: body mass index
CI: confidence interval
PSM: Propensity score matching
RAAS: Renin‐angiotensin‐aldosterone system
RR: risk ratio

## 1 Introduction

Hypertension is a common chronic disease characterized by elevated arterial blood pressure [1]. Approximately 46% of adult patients are unaware that they have hypertension [2]. Hypertension is a risk factor for reduced healthy life expectancy and can further result in heart failure [3], chronic kidney disease [4], retinopathy [5], dementia [6], and other complications. About 1.3 billion adults worldwide have high blood pressure, with the majority residing in low‐ and middle‐income countries [2]. Hypertension is one of the leading causes of death, with an estimated 7.7–10.4 million casualties per year, and disability worldwide [7]. The standardized prevalence of hypertension in China has increased from 20.8% in 2004 to 24.7% in 2018 [8]. Although slight improvements in the rates of treatment and control of hypertension in China have been observed in recent years, these rates are still not optimal [9]. Results from six rounds of national surveys showed that in 2018, 240 million adults with hypertension in China still had poorly controlled blood pressure; of these, 164 million were unaware of their condition, and a further 10 million were not receiving appropriate treatment [8]. In addition, a cox regression analysis in China reported that the prevalence of hypertension increased with age (12.6% in 35–39 years and 58.4% in 70–74 years) [10]. The management of hypertension in older adults should be primarily focused on due to the increasing aging of this population. Physiological lesions [11], lifestyle [12], and genetics [13] are major risk factors for hypertension.

Several studies have confirmed that gout is a substantial risk factor for hypertension [14, 15]. Genetic research has revealed a one‐way relationship between gout and hypertension [14]. A case–control study in the UK used logistic regression to reveal an independent association between gout and hypertension [16]. Another case–control study found that patients with gout had higher body mass index (BMI) levels, with this potentially being linked to hypertension through metabolic pathways. Hyperuricaemia is a prerequisite for gout [17] and contributes to the pathogenesis of hypertension [18]. Cross‐sectional studies in China have shown a positive association of higher serum uric acid levels with hypertension in men [19]. Empirical analyses have indicated that BMI may link these conditions. Gout patients exhibit significantly higher BMIs [20], and population studies have confirmed graded increases in the hypertension risk with BMI elevation [21]. Hyperuricaemia is closely related to adult obesity, and metabolic disorders correlate with weight gain in this population [22]. Being overweight or obese increases the risk of hypertension through documented mechanisms [23]. The higher the BMI, the greater the risk of developing hypertension, underscoring the function of BMI as a modifiable intermediary. There are four main types of hypertension drug treatment: renin–angiotensin–aldosterone system (RAAS), calcium channel blockers, *β*‐blockers, and thiazide or thiazide‐like diuretics [7]. Although hypertension can be effectively controlled by taking medication on time, only 43.7% of patients with hypertension in the United States have controlled their blood pressure due to medication adherence [23]. Because early hypertension exhibits only mild or no symptoms, its harmfulness is often underestimated [19]. Therefore, identifying the optimal targets for hypertension prevention is crucial. Non‐drug therapies that are non‐toxic and easy to administer may be more valuable than drug therapies that are highly toxic and have poor compliance. Dietary therapies and BMI control for gout are becoming increasingly sophisticated, which may provide a necessary solution to hypertension and its complications [17, 24].

Therefore, this study used the cross‐sectional survey data of older adults aged over 65 years in Wuhan, China, in 2020 to explore the relationship between BMI‐mediated gout and hypertension by using mediation analysis for the first time, and further analyzed the impact of BMI and gout on hypertension, hoping to provide critical guidelines for the clinical management of hypertension.

## 2 Methods

### 2.1 Study Participants

The study sample comprised older adults aged 65 years and above with household registration or permanent residence in the Hongshan District, Wuhan City, Hubei Province, who voluntarily participated in the informed consent process. Cluster sampling was performed to recruit participants for this cross‐sectional survey. Sampling began in October 2020, and a total of 33,778 residents aged 65 and above who were registered residents or permanent residents of Wuhan City were surveyed on their physical fitness and health status.

All procedures contributing to this work complied with the ethical standards of the relevant national and institutional committees on human experimentation and with the Helsinki Declaration of 1975, as revised in 2013. All procedures involving human subjects were approved by the Ethics Committee of the Wuhan University of Science and Technology Medical College (ethical approval number 202059).

#### 2.1.1 Inclusion Criteria

1. Participants aged ≥ 65 years who had complete diagnostic information for both gout and hypertension.
2. Permanent residents of Hongshan District, Wuhan, registered in the local health database for ≥ 5 years.
3. Availability of complete baseline data, including BMI, blood pressure measurements, serum uric acid levels, and medication history.
4. No participation in other clinical trials or interventions related to hypertension/gout management within the past 6 months.

#### 2.1.2 Exclusion Criteria

1. Secondary hypertension (e.g., due to renal artery stenosis, hyperaldosteronism) or asymptomatic hyperuricemia without clinical gout.
2. Severe comorbidities affecting BMI or metabolic status (e.g., end‐stage renal disease, decompensated heart failure, active malignancy).
3. Use of systemic corticosteroids, immunosuppressants, or urate‐lowering therapy within the past 3 months.
4. Cognitive impairment or language barriers preventing reliable self‐reported health information.
5. Those who did not cooperate with the study procedures.

### 2.2 Method of Investigation

The older adult subjects received a free physical examination, which primarily included five aspects: personal health information survey, general physical examination, electrocardiogram examination, abdominal B ultrasound examination, and laboratory examination. The physical examination record form was prepared by Wuhan City Health Commission and Wuhan City Centre for Disease Control and Prevention (details of the form can be found in the Appendix S1).

### 2.3 Mediation Analysis With Robustness Assessment

#### 2.3.1 Propensity Score Matching (PSM)

After collecting information via health examination forms, the demographic characteristics of the study participants were analyzed. To mitigate confounding bias, PSM via logistic regression modeling was implemented. The covariates included demographic characteristics (age and gender), socioeconomic factors (education and marital status), and lifestyle parameters (smoking, alcohol intake, and physical activity). Subjects were assigned to the control and treatment groups based on similar propensity scores [25]. Caliper matching was employed to match hypertensive patients with non‐hypertensive subjects at a ratio of 1:1. Caliper matching is a matching method based on the nearest neighbor matching algorithm. This process was carried out using the MatchIt and TableOne packages in R software. The balance of covariates before and after matching was assessed using the standardized mean difference (SMD; a SMD less than 0.1 indicates a good balance).

#### 2.3.2 Primary Mediation Analysis

The main analysis in this study was a mediation analysis. A four‐step analysis was conducted using the “Mediation Association Analysis Framework” and the “Mediation Association” software package [26]. First, the total association model was used to examine the relationship between hypertension and gout. Logistic regression analysis was employed to adjust for confounding factors (educational attainment, physical activity, dietary habits, and smoking status) that remained after PSM. The second step involved a mediation model using linear regression analysis to examine the relationship between BMI and gout, with the same adjustments applied. The third step addressed the direct association model, using logistic regression analysis to examine the relationships between hypertension and gout, and between hypertension, gout, and BMI, with adjustments made. The final step involved bootstrapping with 1000 simulations to estimate the average causal mediation association (ACME) and the average direct association (ADE), as well as their respective 95% confidence intervals (CIs). Among them, BMI was included in the analysis as a continuous variable.

**FIGURE 1:**
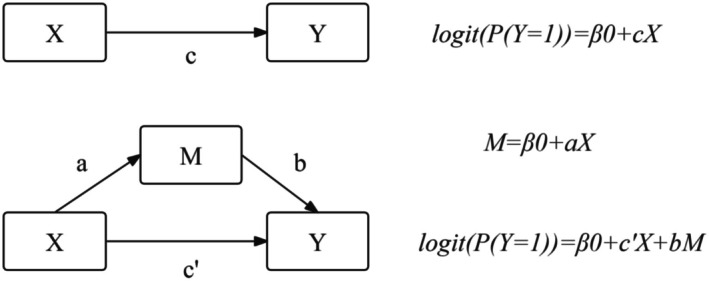
Mediation model path diagram. X represents gout. M represents body mass index. Y represents hypertension. The coefficient *c* is the total association of X on Y. The coefficient *a* is the association of X on M. The coefficient *b* is the association of M on Y after X is controlled. The coefficient *c*′ represents the direct association of X on Y after controlling for M.

Figure 1 shows the path diagram of the mediation model. The product of the coefficients *a* and *b* (*ab*) represents the indirect association of X on Y. The quantitative relationship between the total, direct, and indirect association can be expressed as follows: *c* = *c*′ + *ab*.

After the mediation analysis, bootstrapping was used to statistically test the mediating association, because stepwise regression can only determine the magnitude of the coefficient value of each regression equation and whether the coefficient is significant. Bootstrapping is a resampling method that takes independent samples from the existing sample data and substitutes the same number of samples to make inferences in these resampled data.

#### 2.3.3 Sensitivity Analysis

To assess the robustness of these findings, two sensitivity analyses were performed. Given the known gender differences in the pathophysiology of hypertension, the mediation analysis was performed separately for men and women to assess potential gender‐modified associations. Stratified mediation analyses were conducted by gender using the same four‐step framework. Gender‐specific coefficients and 95% CIs were compared using *z*‐tests for interaction associations. To quantify the potential impact of unmeasured confounders, the e‐values were calculated using the “*E*‐Value” package in R. This analysis estimated the minimum required association strength between unmeasured confounders and the exposure and outcome to fully explain the observed mediating association.

### 2.4 Statistical Analysis

**FIGURE 2:**
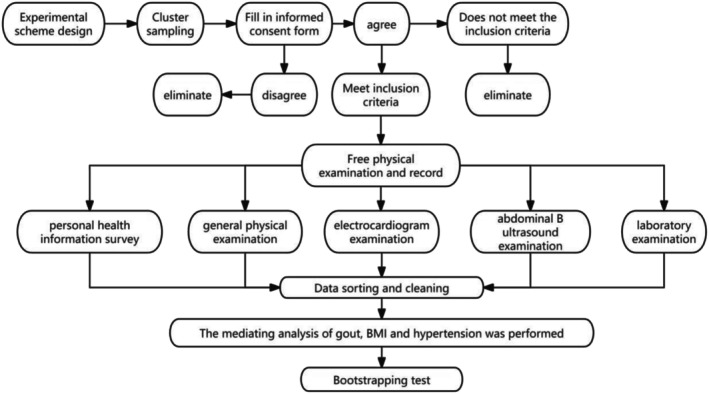
Study flow chart.

The raw data were sorted and cleaned in MS Excel, and subsequent data analysis was conducted using R version 4.3.3. All statistical tests were two‐sided, with a significance level of *α* = 0.05. Continuous variables are presented as the mean ± standard deviation and were compared using *t*‐tests or ANOVA. Categorical variables are reported as frequencies (percentages) and were compared using chi‐squared tests. Model assumptions (linearity and homoscedasticity) were verified using residual diagnostics. The complete analytical workflow is depicted in Figure 2. *p* < 0.050 was considered statistically significant.

## 3 Results

### 3.1 Mediation Analysis With Robustness Assessment

#### 3.1.1 Results of PSM

**FIGURE 3:**
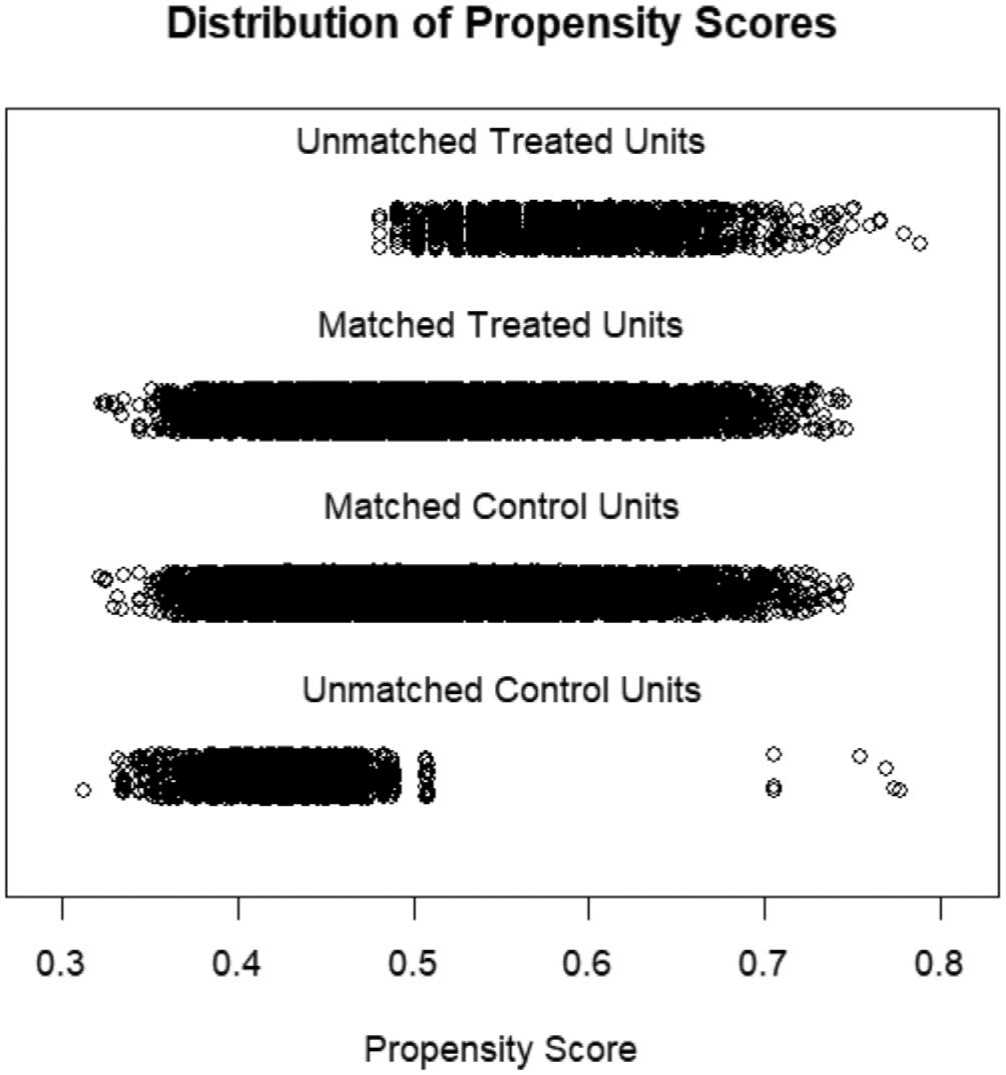
Visualization of propensity score matching results.

The sample comprised 33,778 older adults aged 65 years and above recruited from Wuhan, Hubei Province, in 2020. After sorting and cleaning the data in MS Excel, the items used in this study included hypertension, gout, gender, age, education level, marital status, BMI, exercise, meat and vegetable balanced diet, primarily meat diet, primarily vegetarian diet, addicted to oil, addicted to salt, addicted to sugar, smoking, and drinking. The Appendix S1 comprehensively present the specific distribution of each item. There were 16,680 patients with hypertension and 17,098 people without hypertension, with an average age of 76.30 ± 6.22 and 74.69 ± 5.73 years, respectively. Compared to people without hypertension, patients with hypertension exhibited differences with respect to age, marital status, education level, physical exercise, BMI, meat and vegetable balanced diet, vegetarian diet, addicted to oil, smoking, and drinking (*p* < 0.050) (Table 1). Given these confounding factors (age, marital status, education level, physical exercise, meat and vegetable balanced diet, vegetarian diet, addicted to oil, smoking, and drinking), the PSM method in R language was used for 1:1 matching between people with and without hypertension. Finally, 14,717 pairs were successfully matched. A total of 1963 patients with hypertension and 2381 people without hypertension were not matched. Figure 3 presents a visualization of the PSM (Figure 3). Compared with people without hypertension, after successful matching, patients with hypertension only exhibited differences in six factors: education level, BMI, balanced physical exercise, meat and vegetable balanced diet, vegetarian diet, and smoking (*p* < 0.050, but all SMDs < 0.1) (Table 1).

**TABLE 1:** General data analysis of hypertensive patients and non‐hypertensive people.

		Before propensity score matching		*p*	SMD	After propensity score matching		*p*	SMD
		Non‐hypertensive people	Hypertension patients			Non‐hypertensive people	Hypertensive patients		
Gender, *n* (%)		—	—	0.613	0.006	—	—	0.138	0.017
Male		8258 (48.3%)	8103 (48.6%)	—	—	7158 (48.6%)	7030 (47.8%)	—	—
Female		8840 (51.7%)	8577 (51.4%)	—	—	7559 (51.4%)	7687 (52.2%)	—	—
Age (years, mean ± SD)		74.69 ± 5.73	76.30 ± 6.22	< 0.001*	0.268	75.37 ± 5.83	75.45 ± 5.88	0.244	0.014
Marital status, *n* (%)		—	—	< 0.001*	0.068	—	—	0.062	0.032
Married		14,596 (85.4%)	13,867 (83.1%)	—	—	12,540 (85.2%)	12,410 (84.3%)	—	—
Divorce		144 (0.8%)	120 (0.7%)	—	—	92 (0.6%)	115 (0.8%)	—	—
Widowed		2331 (13.6%)	2671 (16.0%)	—	—	2071 (14.1%)	23,170 (14.7%)	—	—
Unmarried		27 (0.2%)	22 (0.1%)	—	—	14 (0.1%)	22 (0.1%)	—	—
Education, *n* (%)		—	—	< 0.001*	0.082	—	—	< 0.001*	0.097
Primary school or below		4296 (25.1%)	3854 (23.1%)	—	—	3457 (23.5%)	3836 (26.1%)	—	—
Junior high school		4882 (28.6%)	4622 (27.7%)	—	—	4212 (28.6%)	4401 (29.9%)	—	—
Technical secondary school or senior high school		3735 (21.8%)	3540 (21.2%)	—	—	3191 (21.7%)	3190 (21.7%)	—	—
Junior college or above		4185 (24.5%)	4664 (28.0%)	—	—	3857 (26.2%)	3290 (22.4%)	—	—
BMI (mean ± SD)		23.32 ± 3.17	24.69 ± 3.25	< 0.001*	0.428	23.55 ± 3.16	24.63 ± 3.27	< 0.001*	
Gout, *n* (%)		—	—	< 0.001*	0.107	—	—	< 0.001*	0.102
No		16,921 (99.0%)	16,276 (97.6%)	—	—	14,562 (98.9%)	14,368 (97.6%)	—	—
Yes		177 (1.0%)	404 (2.4%)	—	—	155 (1.1%)	349 (2.4%)	—	—
Physical exercise, *n* (%)		—	—	0.003*	0.041	—	—	< 0.001*	0.086
Always exercise		11,554 (67.5%)	11,160 (66.9%)	—	—	9994 (67.9%)	9417 (64.0%)	—	—
Exercise sometimes		738 (4.3%)	831 (4.9%)	—	—	677 (4.6%)	691 (4.7%)	—	—
Exercise occasionally		1002 (5.8%)	1072 (6.4%)	—	—	896 (6.1%)	1058 (7.2%)	—	—
Never exercise		3804 (22.2%)	3617 (21.6%)	—	—	3150 (21.4%)	3551 (24.1%)	—	—
Dietary habit, *n* (%)		—	—	—	—	—	—	—	—
Meat and vegetable balanced	No	1376 (8.0%)	1519 (9.1%)	0.001*	0.038	1250 (8.5%)	1429 (9.7%)	< 0.001*	0.042
	Yes	15,722 (92.0%)	15,161 (90.9%)			13,467 (91.5%)	13,288 (90.3%)		
Meat mainly	No	16,879 (98.7%)	16,470 (98.7%)	0.896	0.002	14,536 (98.8%)	14,518 (98.6%)	0.380	0.011
	Yes	219 (1.3%)	210 (1.3%)			181 (1.2%)	199 (1.4%)		
Vegetarian mainly	No	15,882 (92.9%)	15,333 (91.9%)	0.001*	0.036	13,604 (92.4%)	13,458 (91.4%)	0.002*	0.036
	Yes	1216 (7.1%)	1347 (8.1%)			1113 (7.6%)	1259 (8.6%)		
Addicted to salt	No	16,899 (98.8%)	16,446 (98.6%)	0.057	0.021	14,536 (98.8%)	14,505 (98.6%)	0.128	0.018
	Yes	199 (1.2%)	234 (1.4%)			181 (1.2%)	212 (1.4%)		
Addicted to oil	No	16,993 (99.4%)	16,525 (99.1%)	0.001*	0.036	14,614 (99.3%)	14,595 (99.2%)	0.228	0.015
	Yes	105 (0.6%)	155 (0.9%)			103 (0.7%)	122 (0.8%)		
Addicted to sugar	No	17,015 (99.5%)	16,602 (99.5%)	0.874	0.003	14,639 (99.5%)	14,646 (99.5%)	0.622	0.007
	Yes	83 (0.5%)	78 (0.5%)			78 (0.5%)	71 (0.5%)		
Smoking, *n* (%)		—	—	< 0.001*	0.086	—	—	0.002*	0.042
Never smoked		14,248 (83.3%)	14,190 (85.1%)	—	—	12,478 (84.8%)	12,276 (83.4%)	—	—
Occasionally smoked		720 (4.2%)	832 (5.0%)	—	—	680 (4.6%)	792 (5.4%)	—	—
Often smoked		2130 (12.5%)	1658 (9.9%)	—	—	1559 (10.6%)	1649 (11.2%)	—	—
Drinking, *n* (%)		—	—	< 0.001*	0.055	—	—	0.111	0.029
Never drink		14,248 (83.3%)	14,190 (85.1%)	—	—	12,429 (84.5%)	12,275 (83.4%)	—	—
Occasionally drink		1307 (7.6%)	1226 (7.4%)	—	—	1116 (7.6%)	1191 (8.1%)	—	—
Often drink		255 (1.5%)	205 (1.2%)	—	—	184 (1.3%)	200 (1.4%)	—	—
Drink every day		1288 (7.5%)	1059 (6.3%)	—	—	988 (6.7%)	1051 (7.1%)	—	—

*Note:* *p < 0.050 indicates statistical significance.

Abbreviations: BMI, body mass index; SD, standard deviation; SMD, standardized mean difference.

The general demographic profile of patients with gout was also analyzed. There were 581 patients with gout and 33,197 people without gout before PSM. Compared to people without gout, patients with gout exhibited differences with respect to gender, marital status, education level, BMI, physical exercise, meat and vegetable balanced diet, vegetarian diet, addicted to salt, addicted to oil, smoking, and drinking (*p* < 0.050). There were 504 patients with gout and 28,930 people without gout after PSM. Compared to people without gout, patients with gout exhibited differences with respect to gender, marital status, education level, BMI, physical exercise, meat and vegetable balanced diet, vegetarian diet, smoking, and drinking (*p* < 0.050) (Table 2).

**TABLE 2:** General data analysis of gout patients and non‐gout people.

		Before propensity score matching		*p*	SMD	After propensity score matching		*p*	SMD
		Non‐gout people	Gout patients			Non‐gout people	Gout patients		
Gender, *n* (%)		—	—	< 0.001*	0.687	—	—	< 0.001*	0.687
Male		15,901 (47.9%)	460 (79.2%)	—	—	13,790 (47.7%)	398 (79.0%)	—	—
Female		17,296 (52.1%)	121 (20.8%)	—	—	15,140 (52.3%)	106 (21.0%)	—	—
Age (years, mean ± SD)		75.48 ± 6.03	75.90 ± 6.36	0.099	0.067	75.41 ± 5.85	75.38 ± 5.98	0.917	0.005
Marital status, *n* (%)		—	—	0.006*	0.154	—	—	0.003*	0.169
Married		27,947 (84.2%)	516 (88.8%)	—	—	24,499 (84.7%)	451 (89.5%)	—	—
Divorce		259 (0.8%)	5 (0.9%)	—	—	203 (0.7%)	4 (0.8%)	—	—
Widowed		4944 (14.9%)	58 (10.0%)	—	—	4194 (14.5%)	47 (9.3%)	—	—
Unmarried		47 (0.1%)	2 (0.3%)	—	—	34 (0.1%)	2 (0.4%)	—	—
Education, *n* (%)		—	—	< 0.001*	0.268	—	—	< 0.001*	0.248
Primary school or below		8064 (24.3%)	86 (14.8%)	—	—	7212 (24.9%)	81 (16.1%)	—	—
Junior high school		9330 (28.1%)	174 (29.9%)	—	—	8451 (29.2%)	162 (32.1%)	—	—
Technical secondary school or senior high school		7154 (21.6%)	121 (20.8%)	—	—	6278 (21.7%)	103 (20.4%)	—	—
Junior college or above		8649 (26.1%)	200 (34.4%)	—	—	6989 (24.2%)	158 (31.3%)	—	—
BMI (mean ± SD)		23.98 ± 3.27	25.05 ± 3.36	< 0.001*	0.321	24.07 ± 3.26	25.06 ± 3.35	< 0.001*	0.299
Physical exercise, *n* (%)		—	—	0.007*	0.132	—	—	0.005*	0.145
Always exercise		22,329 (67.3%)	385 (66.3%)	—	—	19,094 (66.0%)	317 (62.9%)	—	—
Exercise sometimes		1543 (4.6%)	26 (4.5%)	—	—	1348 (4.7%)	20 (4.0%)	—	—
Exercise occasionally		2019 (6.1%)	55 (9.5%)	—	—	1901 (6.6%)	53 (10.5%)	—	—
Never exercise		7306 (22.0%)	115 (19.8%)	—	—	6587 (22.8%)	114 (22.6%)	—	—
Dietary habit, *n* (%)		—	—	—	—	—	—	—	—
Meat and vegetable balanced	No	2802 (8.4%)	93 (16.0%)	< 0.001*	0.233	2595 (9.0%)	84 (16.7%)	< 0.001*	0.232
	Yes	30,395 (91.6%)	488 (84.0%)			26,335 (91%)	420 (83.3%)		
Meat mainly	No	32,777 (98.7%)	572 (98.5%)	0.675	0.024	28,558 (98.7%)	496 (98.4%)	0.693	0.025
	Yes	420 (1.3%)	9 (1.5%)			372 (1.3%)	8 (1.6%)		
Vegetarian mainly	No	30,717 (92.5%)	498 (85.7%)	< 0.001*	0.220	26,635 (92.1%)	427 (84.7%)	< 0.001*	0.231
	Yes	2480 (7.5%)	83 (14.3%)			2295 (7.9%)	77 (15.3%)		
Addicted to salt	No	32,779 (98.7%)	566 (97.4%)	0.009*	0.096	28,549 (98.7%)	492 (97.6%)	0.062	0.079
	Yes	418 (1.3%)	15 (2.6%)			381 (1.3%)	12 (2.4%)		
Addicted to oil	No	32,947 (99.2%)	571 (98.3%)	0.016*	0.088	28,713 (99.2%)	496 (98.4%)	0.060	0.078
	Yes	250 (0.8%)	10 (1.7%)			217 (0.8%)	8 (1.6%)		
Addicted to sugar	No	33,041 (99.5%)	576 (99.1%)	0.293	0.048	28,786 (99.5%)	499 (99.0%)	0.217	0.058
	Yes	156 (0.5%)	5 (0.9%)			144 (0.5%)	5 (1.0%)		
Smoking, *n* (%)		—	—	< 0.001*	0.374	—	—	< 0.001*	0.409
Never smoked		28,037 (84.5%)	401 (69.0%)	—	—	24,415 (84.4%)	339 (67.3%)	—	—
Occasionally smoked		1492 (4.5%)	60 (10.3%)	—	—	1416 (4.9%)	56 (11.1%)	—	—
Often smoked		3668 (11.0%)	120 (20.7%)	—	—	3099 (10.7%)	109 (21.6%)	—	—
Drinking, *n* (%)		—	—	< 0.001*	0.339	—	—	< 0.001*	0.378
Never drink		28,028 (84.4%)	410 (70.6%)	—	—	24,359 (84.2%)	345 (68.5%)	—	—
Occasionally drink		2449 (7.4%)	84 (14.5%)	—	—	2229 (7.7%)	78 (15.5%)	—	—
Often drink		442 (1.3%)	18 (3.1%)	—	—	368 (1.3%)	16 (3.2%)	—	—
Drink every day		2278 (6.9%)	69 (11.9%)	—	—	1974 (6.8%)	65 (12.9)	—	—

*Note:* *p < 0.050 indicates statistical significance.

Abbreviations: BMI, body mass index; SD, standard deviation; SMD, standardized mean difference.

#### 3.1.2 Primary Mediation Analysis

The matched data in R were used to conduct a stepwise regression for the independent variable (gout), the mediating variable (BMI), and the dependent variable (hypertension). It is worth noting that after PSM, there were still residual confounding associations with education, physical exercise, diet (meat and vegetable balanced, primarily vegetarian), and smoking. Thus, these variables were included in the mediation model for further adjustment. Stepwise regression analysis revealed that gout was directly associated with hypertension (*β* = 0.733, *p* < 0.010) and strongly associated with BMI (*β* = 1.034, *p* < 0.010). After controlling for BMI, the association between gout and hypertension persisted, suggesting that BMI may function as an intermediary variable in their statistical relationship (Table 3).

**TABLE 3:** Summary of stepwise regression results of mediation analysis.

	Dependent variable		
	Hypertension (1)	BMI (2)	Hypertension (3)
Gout	0.820* (0.098)	1.034* (0.146)	0.733* (0.099)
BMI	—	—	0.104* (0.004)
Constant	0.123 (0.090)	24.287* (0.146)	−2.391* (0.130)
Education	−0.081* (0.011)	−0.191* (0.017)	−0.063* (0.011)
Physical exercise	0.059* (0.009)	0.058* (0.015)	0.055* (0.009)
Meat and vegetable balanced	−0.115 (0.080)	0.128 (0.130)	−0.137 (0.081)
Vegetarian mainly	0.014 (0.085)	−0.100 (0.137)	0.021 (0.086)
Smoking	0.044* (0.018)	0.030 (0.030)	0.041* (0.026)
Observations	29,434	29,434	29,434

*Note:* (1) indicates the total association model, which was used to examine the relationship between hypertension and gout. Logistic regression analysis was employed to adjust for confounding factors (educational attainment, physical activity, dietary habits, and smoking status) that remained after PSM. (2) indicates the mediation model using linear regression analysis to examine the relationship between body mass index and gout, with the same adjustments applied. (3) indicates the direct association model, which used logistic regression analysis to examine the relationships between hypertension and gout, and between hypertension, gout, and body mass index, with adjustments made.

Abbreviation: BMI: body mass index.

* *p* < 0.010.

**FIGURE 4:**
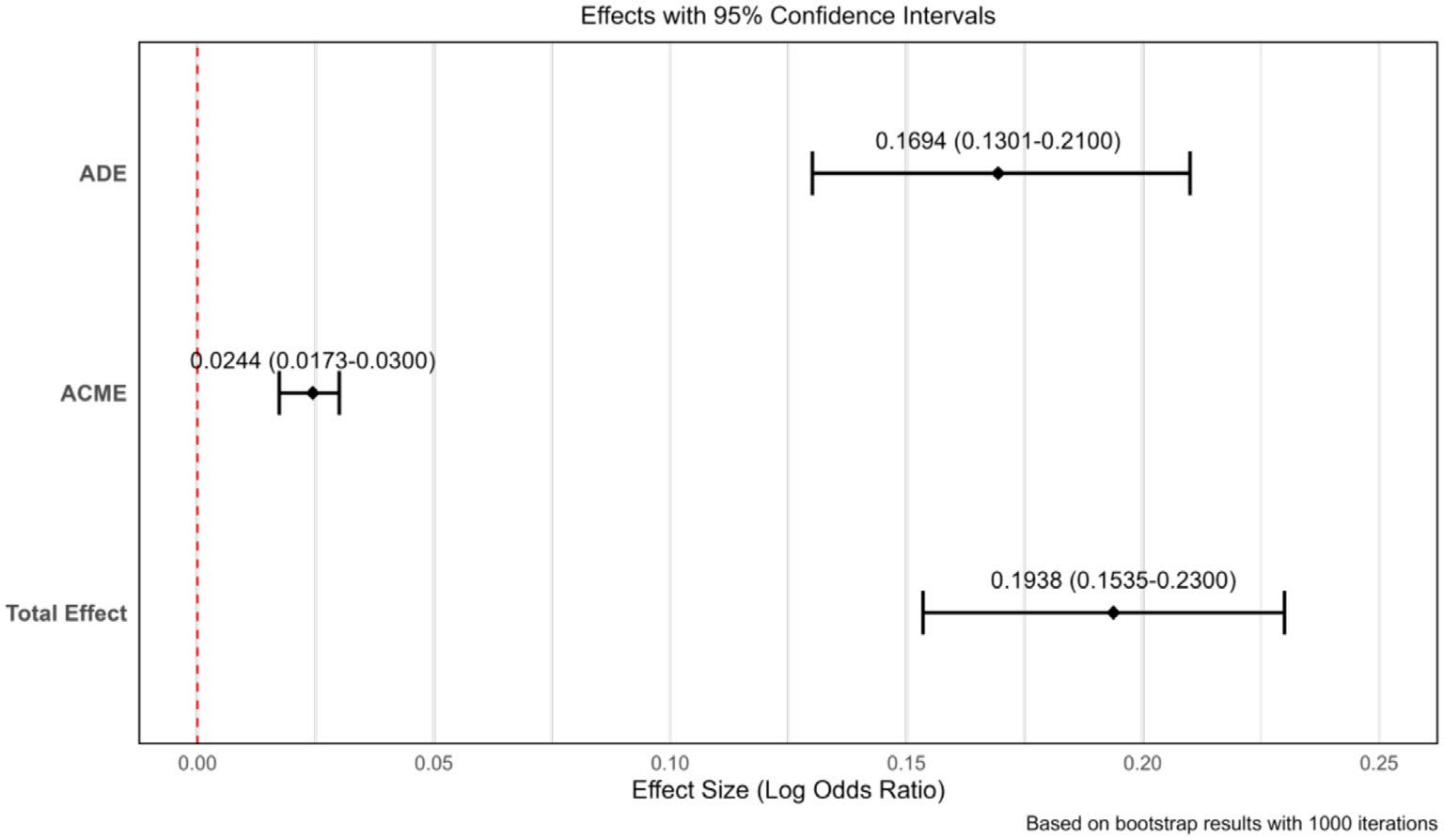
Mediation associations of BMI in the Gout–Hypertension relationship. ACME, average mediation association; ADE, average direct association. ACME/ADE: Model‐derived association metrics under counterfactual framework; not causal estimates.

A bootstrapping analysis with 1000 iterations was performed to assess the stability of the observed statistical associations (Table 4). The ACME value of 0.024 (95% CI: 0.017–0.030) quantifies the indirect association through BMI pathways. The ADE value of 0.169 (95% CI: 0.130–0.210) reflects the association between gout and hypertension that remained after adjusting for BMI. The total association was 0.194 (95% CI: 0.154–0.230), 12.590% (95% CI: 8.630–18.000) of which was potentially attributable to BMI‐mediated pathways. These mediation associations are visualized in Figure 4, including the relative magnitudes of the direct and indirect pathways.

**TABLE 4:** Results of mediation analysis.

Index	Estimate	95% CI lower	95% CI upper	*p*
ACME	0.024	0.017	0.030	< 0.001*
ADE	0.169	0.130	0.210	< 0.001*
Total association	0.194	0.154	0.230	< 0.001*
Prop. mediated (%)	12.590	8.600	18.000	< 0.001*

*Note:* The above model was adjusted for the following confounding factors, which remained statistically significant after PSM: education, physical exercise, balance of meat and vegetables in the diet, vegetarianism, and smoking.

Abbreviations: ACME, average causal mediation association; ADE, average direct association; CI, confidence interval; Prop. mediated, the ratio of the intermediate association to the total association.

* *p* < 0.001.

It is important to note that the ACME and ADE values are model‐based estimates derived under counterfactual assumptions. In this cross‐sectional design, they represent statistical associations rather than demonstrated causal associations, as it is not possible to establish temporal precedence. The narrow confidence intervals (e.g., the ACME range was 0.013) suggest stable statistical patterns in the observed data.

#### 3.1.3 Sensitivity Analysis

**FIGURE 5:**
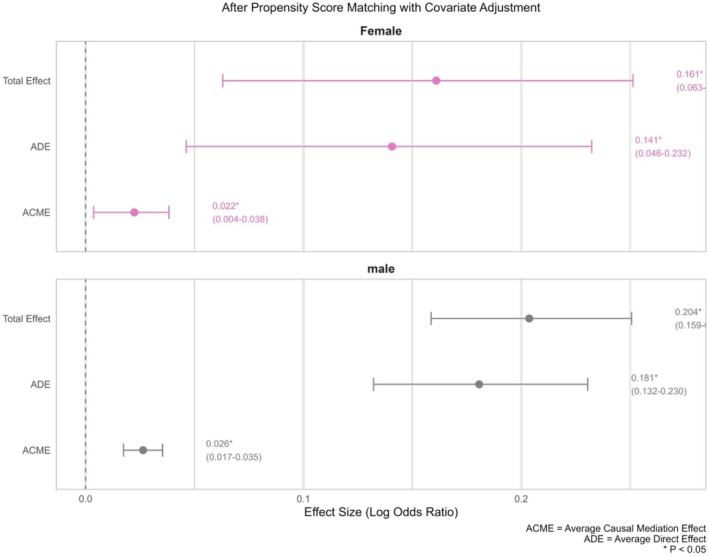
Gender‐stratified mediation associations of BMI in Gout–Hypertension relationship. ACME, average mediation association; ADE, average direct association. ACME/ADE: Model‐derived association metrics under counterfactual framework; not causal estimates.

*E*‐Value analysis revealed an *E*‐Value of 1.44, indicating that the current mediation association results exhibited moderate sensitivity to unmeasured confounding factors. Both the strength of the association between the exposure factor and the outcome variable must reach a risk ratio (RR) ≥ 1.44 to completely overturn the study conclusions. In addition, the robustness of the results for the female group was lower than that for the male group (Table 5 and Figure 5). This suggests that conclusions should be made cautiously, given the potential influence of unmeasured confounding factors.

**TABLE 5:** Results of gender‐stratified mediation analysis.

Gender	Index	Estimate	95% CI lower	95% CI upper	*p*
Male	ACME	0.026	0.017	0.035	< 0.000*
	ADE	0.181	0.132	0.231	< 0.000*
	Total association	0.204	0.159	0.251	< 0.000*
Female	ACME	0.022	0.004	0.038	0.010*
	ADE	0.141	0.046	0.232	0.004*
	Total association	0.161	0.063	0.251	< 0.000*

*Note:* The above model was adjusted for the following confounding factors, which remained statistically significant after PSM: education, physical exercise, balance of meat and vegetables in the diet, vegetarianism, and smoking.

Abbreviations: ACME, average causal mediation association; ADE, average direct association; CI, confidence interval; Prop. mediated, the ratio of the intermediate association to the total association.

* *p* < 0.050.

## 4 Discussion

This study employed PSM and mediation analysis to reveal the intricate statistical relationships between gout, BMI, and hypertension. After adjusting for confounding factors, BMI was found to exhibit the statistical characteristics of a mediating variable in the relationship between gout and hypertension. The indirect association accounted for 12.590% of the variance in the relationship between hypertension and gout. This pattern is consistent with studies on hyperuricaemia. Laboratory studies indicate an association between uric acid exposure and adipocyte inflammatory responses via the activation of the NLRP3 inflammasome [27, 28]. These biological pathways are correlated with the development patterns of hypertension observed in epidemiological studies [29, 30, 31]. It is worth noting that these explanatory mechanisms originate from basic research. Our observational data primarily reveal patterns of association between variables, rather than providing proof of mechanisms.

Additionally, uric acid accumulation in patients with gout may be associated with altered endothelial cell function [32, 33]. A cross‐sectional survey in the United States showed that 84% of patients with hypertension were overweight or obese [23]. From a biological perspective, obesity may be associated with hypertension via pathways such as neurohormonal activation and inflammatory responses [34]. Notably, the increased number of adipocytes in obese individuals may strengthen the statistical association between BMI and hypertension when combined with gout.

The statistical model used in this study indicated that, for every one‐unit increase in the prevalence of gout, the prevalence of hypertension increased by 2.440% through the BMI pathway, with an indirect association value of 0.0244. While this pathway accounted for just 12.590% of the total association, it is noteworthy from a public health perspective, given that BMI is modifiable. Model projections suggest that, among 10,000 older adult gout patients, a 1 kg/m^2^ reduction in BMI could correspond to a 2.440% decrease in hypertension prevalence. While these findings provide a scientific basis for intervention strategies, the nature of observational studies means that their conclusions require further empirical support.

In the context of China's rapidly aging population, the prevalence of hyperuricaemia among people aged 60 years and over is 21% [35], making weight control an integral part of comprehensive management strategies. For overweight or obese older adult patients with gout, BMI intervention can complement conventional treatment. When evaluating these strategies, consideration should be given to the impact of limitations in study design on the generalisability of the conclusions.

From a clinical perspective, two actionable implications emerge. Firstly, dynamic BMI monitoring should be integrated into the hypertension screening system for older adult gout patients. Secondly, dietary interventions should be implemented to synergistically control purine and sodium intake in regions with high salt consumption (such as Hubei) [36]. The practical value of these recommendations needs to be validated through further intervention studies.

In summary, this study offers a fresh approach to the prevention and management of hypertension in older adults by quantifying the role of BMI as a mediator. Combining gout management with cardiovascular care and implementing gender‐specific interventions may have synergistic effects.

## 5 Strengths and Limitations

A feature of this study is the use of the caliper matching method in the PSM. This method addresses the issue of the nearest neighbor matching method not ensuring the matching quality when the matching value distribution gap between the matching group and the treatment group is large.

This study also has some limitations. In this study, some observations may fall outside the caliper value range in variable matching. In the matched demographic data analysis, there are still five factors that differ between patients with hypertension and people without hypertension, which the above operations may cause. In addition, there may be confounding factors that are not measured (such as genetic predisposition, long‐term drug use, renal function, serum uric‐acid concentration, and antihypertensive or urate‐lowering therapy). Thus, more representative data are needed in the future to further validate the results. Furthermore, the dataset for this survey did not have sampling weights, which may limit the generalisability beyond the Hongshan District. Nonetheless, the large sample size and rigorous matching process enhance the internal validity of local public health decision‐making.

## 6 Conclusion

This cross‐sectional study revealed complex associations between gout and hypertension, which may involve BMI as an intermediary. As a rigorously conducted cross‐sectional study, these findings generate valuable hypotheses about the role of metabolic pathways in age‐related hypertension. The identified associations provide a foundation for future longitudinal studies to examine temporal dynamics and potential causal mechanisms.

## Author Contributions

J.C. is responsible for the study concept and design. J.W., J.Z., L.Y., C.M., L.C., G.W., Z.S., W.Z., and C.X. are responsible for data acquisition, analysis, and interpretation. J.W. drafted the manuscript. J.C. supervised and revised the study. All authors contributed to discussions in the manuscript. All authors read and approved the final manuscript.

## Ethics Statement

The study was conducted in accordance with the Declaration of Helsinki, and the study was approved by the Ethics Committees of the Wuhan University of Science and Technology Medical College. The ethical approval number is 202059. Informed consent was obtained from all subjects and/or their legal guardian(s). All methods were performed in accordance with the relevant guidelines and regulations.

## Consent

Informed consent was obtained from the participants for publication of this report and any accompanying images.

## Conflicts of Interest

The authors declare no conflicts of interest.

## Supporting information




**Appendix S1:** agm270049‐sup‐0001‐AppendixS1.zip.

## Data Availability

All data analyzed during this study are included in this published article. Datasets and R codes for PSM and mediation analyses are available to researchers who wish to contact the corresponding authors. The figures and tables in this manuscript were generated using RStudio software and the Biorender platform, the latter's publication licence having been included in the Supporting Information.
