# Supplementary material for: Body Mass Index as a Potential Mediator of the Association Between Gout and Hypertension Among Chinese Older Adults: A Mediation Analysis in a Cross‐Sectional Study
Source: Aging Med (Milton). 2025 Oct 13;8(5):434–46. doi: 10.1002/agm2.70049 (PMC12576584; doi:10.1002/agm2.70049)
Supplement: Supplementary file 1 — Appendix S1: agm270049‐sup‐0001‐AppendixS1.zip. [file AGM2-8--s001.zip › Supplementary materials/2020 Physical Examination Register for the elderly.docx]

serial number (tens digit ): 20- □□□□□□□□

International Standard Book Number : □□□□□□- □□□- □□□- □□□□□

**Free physical examination for people over 65 years old in Wuhan in 2020**

**Medical examination book**

Name ：_____________________________

Address ：_____________________________

P hone ：_____________________________

Affiliated primary medical institution ：_____________________________

Physical examination time ：_____________________________

**Wuhan Municipal Health Commission**

**Wuhan Center for Disease Control and Prevention**

**authorized strength**

**Personal health-related information**

| Basic situation | name |  | | | | | | gender | ○male ○female | | | | | date of birth | |  | |
| --- | --- | --- | --- | --- | --- | --- | --- | --- | --- | --- | --- | --- | --- | --- | --- | --- | --- |
|  | ID number |  | | | | | | | current address | | |  | | | | | |
|  | marital status | ○married  ○divorce d  ○widowed  ○unmarried | | | | | | | degree of education | | | ○Primary and below ○J unior high school  ○Secondary school or high school  ○College or above | | | | | |
|  | fix phone* |  | | | | | | | cell phone number | | |  | | | | | |
|  | linkman* |  | | | | | | | contact number* | | |  | | | | | |
|  | way of old-age care | ○Home  ○Nursing institution | | | | | | | | | | | | | | | |
|  | Who takes care of your daily life? | | | | | | ○alone ○husband or wife ○children ○nanny ○other people | | | | | | | | | | |
|  | family history | | | ○No ○Yes ： □hypertension □diabetes □Stroke □coronary heart disease  □hyperlipemia □ Cancer □ asthma □Alzheimer's disease □ other | | | | | | | | | | | | | |
|  | allergic history* | | | ○ No ○ Yes: □ drug □ food □ other | | | | | | | | | | | | | |
| health condition | 1. Are you satisfied with your current health status? | | | | | ○ Satisfied ○ basically satisfied ○ said not clear ○ not very satisfied ○ not satisfied | | | | | | | | | | | |
|  | 2. Have you been diagnosed by the hospital with the disease shown in the bottom? | | | | | | | | | ○ I do not suffer from any disease ○ yes ("yes" need to answer the 3-10 questions) | | | | | | | |
|  | 3. High blood pressure | | Date of diagnosis: Are you taking your medication as prescribed?: ○ Yes ○ No  Have you missed, stopped, or reduced your dose?○ yes, months ○ No | | | | | | | | | | | | | | |
|  | 4. Diabetes | | Date of diagnosis: Are you prescribed medication:  Have you missed, stopped, or reduced your dose? | | | | | | | | | | ○ yes  ○ yes | | ○ no  months | | ○ no |
|  | 5. Stroke | | □ Cerebral ischemia (cerebral infarction), How many times? What year and what month did it happen? When was the last attack?  □ Cerebral hemorrhage (cerebral hemorrhage), How many times? What year and what month did it happen? When was the last attack?  □ Transient cerebral ischemia (TIA), How many times? What year and what month did it happen? When was the last attack?  □ subarachnoid hemorrhage, How many times? What year and what month did it happen? When was the last attack? | | | | | | | | | | | | | | |
|  | 6. Heart disease | | □ myocardial infarction, How many times? What year and what month did it happen? When was the last attack?  □ coronary heart disease □ angina □ coronary revascularization □ congestive heart failure □ pain in the precardiac area | | | | | | | | | | | | | | |
|  | 7. Kidney disease | | □diabetic nephropathy crenal failure □ acute nephritis □ chronic nephritis □ kidney stones | | | | | | | | | | | | | | |
|  | 8. Vascular disease | | □ dissecting aneurysm □ arterial occlusive diseases | | | | | | | | | | | | | | |
|  | 9. Eye disease | | □ cataract glaucoma □ glaucoma □ retinal hemorrhage or exudation □ papilledema | | | | | | | | | | | | | | |
|  | 10. Other | | □ old chronic bronchitis □ asthma □ hyperlipidemia □ prostate disease □ bone and joint disease □ fatty liver□ Gout □ gallstones □ neurological disease □ others | | | | | | | | | | | | | | |
| symptom | □ asymptomatic □ headache □ dizziness □ palpitations □ chest tightness □ chest pain □ chronic cough □ expectoration  □dyspnea □ polydipsia □ polyuria □ weight loss □ fatigue □ joint swelling and pain □ blurred vision □ numbness of hands and feet □urgent urination □ urine pain □ constipation □ diarrhea □ nausea and vomiting □ dizziness □ tinnitus □ breast pain □ others | | | | | | | | | | | | | | | | |
| Diet and exercise | 1. Eating habits | | | | □ Meat and vegetable balance □ meat-based □ vegetarian □ halophilic □ oleophilic □ sugar addiction | | | | | | | | | | | | |
|  | 2. Your average weekly exercise status: | | | | ○ Never exercise ○ occasionally ○ sometimes (1-2 times a week) ○ often (3-5 times a week) ○ Always (6~7 times a week)  (if "never exercise", do not need to fill in 3-5 questions) | | | | | | | | | | | | |
|  | 3. What kind of exercise do you do? | | | | □ Walking □ fast walk □ jogging □ square dance □ kicking shuttlecock □badminton □ swimming □ Tai Chi □ community fitness equipment □ cycling □gyro □ soft force ball □ others | | | | | | | | | | | | |
|  | 4. Your each exercise time: | | | | minutes | | | | | | 5. How many years of exercise? | | | | years | | |

| Smoking status | 1. Description of your current smoking status: | | | ○ never ○ occasionally ○ often ○ have quit smoking  (if "never", do not need to fill in 2-4 questions, if "have quit smoking", fill in 2-4 questions according to the situation before quitting smoking) | | | | | | | | |
| --- | --- | --- | --- | --- | --- | --- | --- | --- | --- | --- | --- | --- |
|  | 2. The average number of cigarettes smoked per day is: | | | cigarettes | | | | | | | | |
|  | 3. Age of the smoking initiation | | | years old | | 4. Age of smoking cessation | | | | | yeas old | |
|  | 5. Mean weekly passive smoking status: | | | ○ No ○ 1~2 days ○ 3~4 days ○ 5~6 days ○ 7 days  (refers to "secondhand smoke") | | | | | | | | |
| Drinking alcohol situation | 1. The description of your current drinking situation is as follows: | | | ○ Never ○ occasionally ○ often ○ daily ○ abstinence (abstinence age: years old)  (If "never", you do not need to fill in 2-5 questions, if "quit drinking", fill in the following questions according to the situation before you quit drinking) | | | | | | | | |
|  | 2. The most common types of alcohol you drink are: | | | ○ Liquor ○ yellow rice wine ○ red wine ○ beer ○ others | | | | | | | | |
|  | 3. The average amount of alcohol consumed per day is: | | | ounce (converted into liquor, 2 bottle of beer, a jin of yellow rice wine, 8 two red wine equivalent to 2 ounce liquor) | | | | | | | | |
|  | 4. The age when you started drinking | | | years old | | 5. Have you been drunk in the past year? | | | | | ○ yes ○ no | |
| harm | 1. Have you ever been hurt in the past year? | | | ○ No ○ Yes (if "no", it is not necessary to fill in 2-4 questions. If there are many times, fill in 2-4 questions according to the most serious situation) | | | | | | | | |
|  | 2. What are the types of injuries? | | | □ fall □ motor vehicle accident □ non-motor vehicle accident □ burn and scald □ sharp device injury□ poisoning □ animal injury □ crash / crush □ others | | | | | | | | |
|  | 3. Where does the injury occur? | | | ○ home ○ public living places ○ roads ○ sports and sports venues ○ others | | | | | | | | |
|  | 4. Nature of the injury (choose the most serious one) | | | ○ fracture ○ sprain / strain ○ blunt trauma, bite, open injury ○ contusion / scratch ○ burn ○ Other | | | | | | | | |
| harmful factor | 1.What are the hazards you have been exposed to at work? | | ○ No ○ (type Practice time: years old)  Poison species: | | | | | | | | | |
|  |  |  | dust:  radiogen: physical factor:    chemical substances:    other: | | | | | | protective measures protective measures protective measures  protective measures  protective measures | | ○yes ○no  ○yes ○no  ○yes ○no  ○yes ○no  ○yes ○no | |
|  | 2. What are the hazards you have been exposed to in your life? | | □ No □ lampblack □ powder dust □ poison carcinogen □ high temperature  □ low temperature □ noise, vibration □ radiation | | | | | | | | | |
| Hospitalization* | history of hospitalization | admission date/  discharge date | | | | | cause | name of medical institution | | | | patient's identification number |
|  |  |  | | | | |  |  | | | |  |
|  |  |  | | | | |  |  | | | |  |
|  | family bed history | date of bed construction/date of bed withdrawal | | | | | cause | name of medical institution | | | | patient's identification number |
|  |  |  | | | | |  |  | | | |  |
|  |  |  | | | | |  |  | | | |  |
| Medication status* | Drug name | usage | | | dosage | | | medication time | | adherence to medication  1. regular 2. intermittent  3. do not take medication | | |
|  |  |  | | |  | | |  | |  | | |
|  |  |  | | |  | | |  | |  | | |
|  |  |  | | |  | | |  | |  | | |
|  |  |  | | |  | | |  | |  | | |
| Vaccination* | name | Date of inoculation | | | Vaccination institutions | | | | | | | |
|  |  |  | | |  | | | | | | | |
|  |  |  | | |  | | | | | | | |
|  |  |  | | |  | | | | | | | |

| Self-care ability | Evaluation items, content, and score | Degree level | | | | |
| --- | --- | --- | --- | --- | --- | --- |
|  |  | Can take care of yourself | Mild dependence | Moderate dependence | Can't take care of yourself | Judgment score |
|  | (1) Eating: use tableware to send food to the entrance, chewing, swallowing and other activities | completed independently (0 points) | (0 Points) | Need assistance, such as chopping, stirring food (3 points) | Complete help required (5 points) |  |
|  | (2) washing: combing hair, wash your face, brush your teeth, shaving and bath and other activities | completed independently  (0 points) | ability to wash hair, wash hair, brush teeth, shave, etc.; bath assistance (1 point) | Part of groactivity with assistance and appropriate time (3 points) | Complete help required (7 points) |  |
|  | (3) Clothing: dressing pants, socks, shoes and other activities | independently accomplish  (0 Points) | —  (0 Points) | Need assistance at the appropriate time (3 points) | Complete help required (5 points) |  |
|  | (4) Toilet: urination, stool and other activities and self-control | no assistance, be self controlled  (0 points) | Incurrent incontinence, But they can basically go to the toilet or use a toilet (1 point) | Frequent incontinence, and they are able to go to the toilet or use toilets with a lot of prompting and assistance. (5 points) | Complete incontinence, totally need for help (10 points) |  |
|  | (5) Activities: standing up, walking indoors, going up and down the stairs, and outdoor activities | complete all activities independently (0 points) | Standing, walking, going up and down stairs with the help of small external force or auxiliary devices (1 point) | With the help of a large external force to complete the standing, walking, can not go up and down the stairs (5 points) | Bedridden, activity needs help (10 points) |  |
|  | Total score | | | | |  |
|  | Self-assessment of self-care ability | ○ Can be self-care (0~3 points) ○ mild dependence (4~8 points) ○ moderate dependence (9~18 points) ○ unable to take care of themselves (19 points) | | | | |

Note: The one marked with "*" in the upper right corner of the project is selected, and the others are required

Survey time: doctor:

**Physical examination results**

| General examination doctor: | | | |
| --- | --- | --- | --- |
| blood pressure | left side / mmHg | | ○ Before taking medicine  ○ After taking medicine  (Note: higher blood pressure than normal should be measured on both sides) |
|  | right side / mmHg | |  |
| Stature: cm (cm) | | | |
| Weight: kilogram (kg) | | | |
| Waistline: centimeter (cm) BMI: Kg /m2 | | | |
| Temperature: ℃ breathe: time / minute | | | |
| PR : time / minute | | | |
| Cognitive and motor function examination: | | | |
| Rough screening of cognitive function | Step 1: The doctor names the three items: 、 、  Step 2: The elderly repeat immediately ○ Can repeat correctly ○ Can not repeat correctly  Step 3: repeat after 1 minute ○ Repeat correctly ○ Can not repeat correctly | | |
| Emotional state coarse sieve | Ask the elderly, " how are you   1. good 2. not very good | ”feeling recently?  3.very bad | |
| Athletic ability test | Step 1: Put your hands on the back of your head.  1. can 2. can’t  Step 2: Pick up items on the ground 1. can 2. can’t  Step 3: Stand up from the chair, turn around, and sit down 1.can 2.can’t | | |
| Agility and Balance test * | Stand up and walk 5 meters (2.5 meters round trip) seconds  *Sit on the chair, start the time, stand up, walk forward 2.5 meters, bypass the markers (benchmarking and other objects), walk 2.5 meters back to the chair, sit down, end the time (note: the elderly who can not stand alone, or walk alone obstacles do not do this, the result is filled "0")* | | |
| Internal medicine examiner: | | | |
| heart rate: times/ cent | | | |
| Heart rhythm: 1. homogeneity 2. arrhythmia 3. definitely out of whack | | | |
| cardiac souffle: 1.no 2. yes | | | |
| barrel chest: 1. no 2. yes | | | |
| Breathing tone: 1. normal 2. abnormal | | | |
| Rale: 1. no 2. dry rale 3. moist rales 4. other | | | |
| abdominal tenderness : 1. no 2. yes | | | |
| Abdominal mass: 1. no 2. yes | | | |
| Hepatomegaly: 1. no 2. yes | | | |
| Splenomegaly: 1. no 2. yes | | | |
| Abdominal mobility dullness: 1. no 2. yes | | | |

| The Surgical Examiner is: | | | | | |
| --- | --- | --- | --- | --- | --- |
| skin | 1. normal 2. flushing 3. pale 4. cyanosis 5. yellow dye  6. hyperpigmentation 7. others | | | | |
| Superficial lymph node: 1. unreachable 2. supraclavicular 3. axilla 4. others | | | | | |
| Lower extremity edema: 1. without 2. unilateral 3. bilateral asymmetry 4. bilateral symmetry | | | | | |
| Foot back artery pulsatile | | | 1. unreachable 2. touched the bilateral symmetry  3. touch left side weak or disappeared 4. touch right side weak or absent | | |
| Ophthalmic and Hearing examination Examphysician: | | | | | |
| uncorrected visual acuity: (left )/ (right) | | | | | (Note: Select 1 item for naked eye and corrected vision. Log vision and "0" for blindness.) |
| corrected visual acuity: (left)/ (right) | | | | |  |
| fundus*  (Non-free item, optional) | | 1. normal 2. abnormal  If abnormal, the abnormal result is: (optional)   1. Diabetic retinopathy 2. Cataract (lens degeneration)   5) Vitreous opacity (floaters)  7) Vascular diseases (vascular arteriosclerosis)  8) others | | 2) Macular disease  4) Glaucoma  6) Optic nerve disease | |
| Sclera: 1. normal 2. icteric 3. congestion 4. other | | | | | |
| Hearing: 1. hearing 2. inaudible | | | | | |
| Stomatology examination examination doctor: | | | | | |
| Lip: 1. ruddy 2. pale 3. cyanosis 4 .chapped 5. herpes | | | | | |
| dentition | 1. normal 2. missing teeth 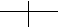 3. dental caries 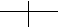  4. denture (denture)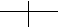  (Note: The specific conditions of tooth loss, dental caries and denture should be filled in the paper rant, and the number of teeth only needs to be recorded into the input system) | | | | |
| pharyngeal : 1. no congestion 2. congestion 3. lymphoid follicle hyperplasia | | | | | |
| Information or remarks to be explained: (if one of the above items refuses to check, please explain and sign by the elderly)  Reject test items: □ questionnaire survey  □ general examination □ cognitive and motor function examination □ internal medicine □ surgery □ ophthalmology and hearing examination □stomatology  Examination of the signature of the elderly: | | | | | |

**ECG test results**

| Conclusion of the ECG test:  1. Normal  2. Abnormal  If abnormal, the abnormal result is: (optional)  1) Early beat (including early chamber, early chamber, and prephase contraction)  2) Excessive slow sinus heart rate  3) Sintral heart rate is uneven or too speed  4) ST_T, wave anomaly (including ST or T-wave anomaly, infarction, infarction, etc.)  5) Conduction block  6) QRS abnormal wave (including high voltage, electrical axial bias, hypertrophy, QRS, etc.)  7) Other    3. This item was not tested (elderly signature:) |
| --- |

**Abdominal B-ultrasound results**

| Conclusion of abdominal B-ultrasound (liver, bile, spleen and pancreas):  1. Normal  2. Abnormal  If abnormal, the abnormal result is:  1) fatty liver 2) liver cyst 3) schistosoma liver 4) hepatic hemangioma  5) gallstones, 6) cholecystitis, 7) gallbladder polyp 8) cholecystectomy  9) Other  3. This item was not tested (elderly signature:) |
| --- |

**Laboratory test results**

| Information or remarks to be explained: (if one of the above items refuses to check, please explain and sign by the elderly)  Reject items: □ blood routine □ urine routine □ biochemical examination  (signature of the elderly: ) |
| --- |

| Conclusion and suggestion of physical examination  Paste place |
| --- |

**conclusion and suggestion**

9
