# Supplementary material for: Body Mass Index as a Potential Mediator of the Association Between Gout and Hypertension Among Chinese Older Adults: A Mediation Analysis in a Cross‐Sectional Study
Source: Aging Med (Milton). 2025 Oct 13;8(5):434–46. doi: 10.1002/agm2.70049 (PMC12576584; doi:10.1002/agm2.70049)
Supplement: Supplementary file 1 — Appendix S1: agm270049‐sup‐0001‐AppendixS1.zip. [file AGM2-8--s001.zip › Supplementary materials/Ethical approval statement.pdf]

Medical Ethics review form of Wuhan University of Science and Technology

Application Date:2020.06.01 Ethical Approval No（202059）

Title: Body Mass Index as a Potential Mediator of the Association between Gout and Hypertension among Chinese Older Adults: A Mediation Analysis in a Cross-Sectional Study
Project start and end time：2020.06~2021.12
Project Types： A. Investigational New Drug B. New technology application<br>C √. Human specimen collection D. Others
The type of review requested： A.√ Apply for projects B. Project after approval<br>C. Continuation of the project D. Authorized project
Research content and significance：<br>**Research content**<br>This study examines whether body mass index (BMI) mediates the association between gout and hypertension, utilising data from elderly individuals aged 65 and above in Wuhan, China. Participants will be enrolled according to inclusion and exclusion criteria, undergoing complimentary health examinations comprising five components: personal health information surveys, general physical examinations, electrocardiograms, abdominal ultrasound scans, and laboratory tests. Baseline data will be analysed using propensity score matching and mediation analysis to validate BMI's mediating role in the relationship between gout and hypertension.<br>**Research significance**<br>① Reveals BMI's mediating mechanism between gout and hypertension, supporting the biological plausibility of metabolic pathways.<br>② Provides epidemiological evidence for the "obesity-hypertension" association, confirming obesity influences blood pressure via neurohormonal activation pathways.<br>③ Confirms BMI as a modifiable factor, offering non-pharmacological intervention directions for elderly gout patients.<br>④ Provides cost-optimised strategies for cardiovascular disease prevention in ageing societies (e.g., weight management as partial substitute for drug therapy).
Abstract of human specimen or human experimental research program：<br><br>This study involves only a cross-sectional screening of participants' basic health status and will not adversely affect their health. All participants will sign an informed consent form prior to taking part in the survey. This project will protect the privacy of participants and their families in accordance with legal requirements. Samples and information will be identified by research identification numbers rather than names.<br><br>Signature of project Leader: Jing Cheng 2020.06. 01

The applicant promise

The research content does not pose harm or risk to participants. Recruitment shall be conducted entirely on the basis of voluntary participation and informed consent, with utmost protection afforded to participants' privacy. This research project complies with the principles of the Declaration of Helsinki and relevant Chinese policies and regulations. I hereby undertake to conscientiously fulfil all relevant obligations and fully respect the rights and interests of research participants.

Signature of project Leader: Jing Cheng 2020.06.01

Review comments：

In this study, the rights and interests of the subjects will be fully protected and there is no potential risk for the subjects. The application for this study is agreed. If the funding is obtained, the on-site work can be carried out as planned.

Signature of project Leader: Jing Cheng . 2020.06.01
