# Supplementary material for: Body Mass Index as a Potential Mediator of the Association Between Gout and Hypertension Among Chinese Older Adults: A Mediation Analysis in a Cross‐Sectional Study
Source: Aging Med (Milton). 2025 Oct 13;8(5):434–46. doi: 10.1002/agm2.70049 (PMC12576584; doi:10.1002/agm2.70049)
Supplement: Supplementary file 1 — Appendix S1: agm270049‐sup‐0001‐AppendixS1.zip. [file AGM2-8--s001.zip › Supplementary materials/Evidence of Language Review.pdf]

NATIVE English Editing
https:www.nativeee.com
Address: 18 East Jiuxianqiao Road,
Chaoyang District, Beijing, China
Phone: +861064125081

16th August, 2025

STATEMENT OF EDITING

This is to certify that the following document has been checked and corrected for proper English language, grammar, punctuation, spelling, and overall style by one or more of the highly-qualified, native English-speaking editors at Native English Editing.

Native English Editing provides editing and proofreading of scientific manuscripts for submission to peer-reviewed journals.

Manuscript title: Body Mass Index as a Potential Mediator of the Association between Gout and Hypertension among Chinese Older Adults: A Mediation Analysis in a Cross-Sectional Study

Date Issued: 16th August, 2025

Certificate Verification Key: 2024060654521005

Yours truly,

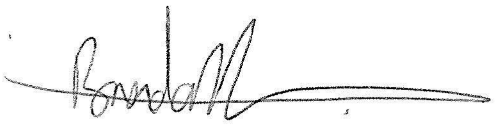

Graeme Brown
Co-owner Native English Editing
Email: publish@nativeee.com
Contact information of Beijing sales department in China:
Address: 18 East Jiuxianqiao Road, Chaoyang District, Beijing, China
Phone: +861064125081
Fax: +861064125081
Contact information of Australian editorial department:
Address: 42D Melrose Street, Parkdale Vic 3195, Australia
Phone: +61417560758
